# Modeling of Years of Cohabitation, Children Ever Born, and Number of Living Children of Women of Reproductive Age Using Trivariate Poisson Regression

**DOI:** 10.1002/hsr2.72982

**Published:** 2026-08-16

**Authors:** Kassim Tawiah, Nana Kena Frempong, Atinuke Olusola Adebanji, Richard Kwame Ansah

**Affiliations:** ^1^ Department of Statistics and Actuarial Science Kwame Nkrumah University of Science and Technology Kumasi Ghana; ^2^ Department of Mathematics Saveetha School of Engineering (SIMATS), Thandalam Chennai Tamil Nadu India; ^3^ Department of Mathematics and Statistics University of Energy and Natural Resources Sunyani Ghana

**Keywords:** cohabitation, expectation‐maximization algorithm, fertility rate, health intervention, pregnancy loss, trivariate Poisson regression, women

## Abstract

**Background and Aims:**

To have the right policies and treatments, population and public health research must comprehend the interrelated dynamics of different cohabitation and reproduction patterns. The effects of cohabitation duration and reproductive experiences on child survival among women of reproductive age are examined in this study.

**Methods:**

Employing a trivariate Poisson regression (TPR) model, the analysis captures the interrelated nature of births within the last 5 years, pregnancy losses, and birth registration. Parameter estimation was conducted using the Expectation‐Maximization algorithm, which is well‐suited for handling complex statistical frameworks. Data were drawn from the 2022 Demographic and Health Survey.

**Results:**

The study indicates significant constant covariance among the three outcomes, confirming their interconnectedness. The findings show that the socio‐demographic factors exert a strong influence on reproductive histories and highlight the pathways through which reproductive experiences affect fertility behavior and child survival. The methodological framework demonstrates improved statistical efficiency and model fit, as evidenced by the likelihood ratio test, Akaike information criterion (AIC), and Bayesian information criterion (BIC).

**Conclusion:**

Importantly, the analysis reveals that cohabitation duration and fertility patterns are strongly shaped by recent pregnancies, pregnancy losses, and birth registration, while child survival is most contingent upon pregnancy losses and registered births. These results emphasize the need for reproductive health policies that provide targeted support for women experiencing recent pregnancies, pregnancy loss, and unregistered births to strengthen child survival outcomes.

## 1 Introduction

It is necessary to understand the interconnected dynamics of various cohabitation and reproductive patterns in population and public health studies to have appropriate policies and interventions [1, 2]. Reproductive behavior of women is essential in formulating policies regarding population control, motherhood, and family planning [3, 4]. Socio‐demographic factors such as years of cohabitation (CHD), total number of children (TCB), and number of living children (NLC) play an important role in women's reproductive history and overall health [5, 6, 7].

CHD refers to the duration in years since a woman of reproductive age (typically 15–49 years) began living with a partner in a marriage‐like relationship, regardless of whether the union is formally married or consensually cohabiting [8]. It plays critical roles in analyzing trends related to family formation, birth spacing, and contraceptive use relative to the length of the relationship. Furthermore, it provides a standardized measure of relationship duration for comparative analysis across different countries and cultures, where the legal definition or prevalence of formal marriage versus informal cohabitation may vary significantly. For women of reproductive age, CHD helps researchers understand how the length of union relates to reproductive outcomes (like fertility rates within marriage) and social indicators (such as decision‐making power or health service use). Fertility rates can be tabulated by duration since cohabitation to approximate marital fertility over time [8].

TCB of women of reproductive age is the total number of live births a woman has ever had in her lifetime, regardless of whether the children are currently alive or deceased. This includes children who are still alive at the time of the survey and children who may have died after birth. It represents the lifetime fertility experience of women up to the time of the survey and is an essential input for measures such as age‐specific fertility rates and total fertility rate when combined with birth event data. This is the true count of children ever born to the woman and so represents a woman's complete lifetime fertility. It is essential for fertility analysis [8].

NLC is the total count of a woman's biological children who are alive at the time of the survey. The count includes all of a woman's biological children, regardless of where they currently live (i.e., at home or elsewhere). For currently pregnant women, the current pregnancy is often included in the count of living children when calculating certain statistics, such as those related to the ideal number of children. The NLC does not include children who were born alive but have since died. It differs from TCB, which counts all births regardless of whether the children are alive, and focuses solely on surviving children. This measure is widely used in analyses of current fertility and child survival, reproductive behavior, and demographic outcomes [8].

These three measures are correlated and count‐based in nature, and therefore, a model design that would capture their common distribution and interrelatedness is needed [9]. While studies usually examine such factors separately, their inherent interconnectedness makes a concurrent modeling approach necessary to ascertain their real dynamics. Three major demographic variables for women are highlighted in this study: years of cohabitation, total number of children, and number of living children. These measures are rudimentary indicators of reproductive health, family formation patterns, and household structure, all of which are influenced by socio‐economic, cultural, and healthcare situations prevalent in Africa and other continents [3, 5, 6, 7].

Increased periods of cohabitation escalate the exposure of women to a higher risk of pregnancy, hence directly influencing the total number of children born by the woman [10, 11, 12, 13]. CHD influences reproductive exposure length, hence increasing the possibility of higher parity [14, 15]. TCB is a measure of fertility that is influenced by socio‐cultural as well as biological factors [16, 17]. NLC measures fertility as well as mortality of children and is influenced by socio‐economic status, maternal age, and availability of health care [18, 19]. NLC is a subset of the total number of children, and the difference between them accounts for the child mortality experience [16, 17]. There can be direct causal links, feedback, and two‐way effects. The present count of surviving children could influence a woman's preference for additional children or the use of contraceptives, which would impact future fertility [20].

Once more, the loss of a child could influence future desires for reproduction [1, 2, 4]. One distinguishing characteristic of this current model is the acknowledgment of unobserved heterogeneity [20]. This is due to the unmeasured characteristics of women or their environment that all influence CHD, TCB, and NLC. These unobserved variables, for example, number of births in the last 5 years, number of pregnancy losses, unmeasured aspects of family planning effectiveness, number of entries in the birth register, or shared community‐level influences, are not normally accounted for in observed covariates. This potentially create correlation between the three outcomes (CHD, TCB, and NLC) that is explained by the observed covariates. Conventional univariate approaches to analyzing such outcomes can miss important dependencies, resulting in incomplete knowledge about their determinants [20].

The total number of children a woman has is directly correlated with how exposed she is to being pregnant, which frequently is associated with her cohabitation length [20]. Similarly, surviving children are a function of children ever born, and also an indication of child mortality experiences, and these can be influenced by fertility and cohabitation [10]. Fertility, cohabitation, and child survival have been topics of research in demography for decades [1, 2, 4].

Researchers have considered these factors in isolation in numerous studies, whereas increasingly more literature is recognizing the importance of their inter‐dependencies. Evidence repeatedly indicates a robust association between the duration of cohabitation and fertility history. Studies in various settings, including those conducted in sub‐Saharan Africa, have found that longer periods of cohabitation are typically associated with higher parity due to increased exposure to the risk of pregnancy [21, 22, 23]. The pattern can be complex, varying according to age at cohabitation, marital status, and contraception use. In sub‐Saharan Africa, cultural marriage patterns and shifting cohabitation norms likely play a significant role in shaping fertility trajectories [24]. The gap between TCB and NLC is a direct indicator of child mortality [25].

Higher levels of child mortality, particularly in the developing world, play a significant role in influencing family size choices and intra‐household resource allocation [26]. Measures such as health care access, maternal education, wealth status, and sanitation are known determinants of child survival. Investigation of the dynamics between TCB and NLC helps in the understanding of population health and in monitoring progress toward child survival goals [8]. Univariate analysis of these outcomes provides valuable information but does not usually display the rich interplay among the demographic outcomes.

Background work in the form of extensive research on fertility and family planning has been available [3, 5, 6, 7, 8]. These studies have typically focused on a single facet or bivariate relationship, leaving a void regarding the overall interplay among these important demographic processes [20, 25, 27]. Joint modeling has been perceived to be in demand across numerous disciplines. For count data, Poisson regression is a common technique [28]. However, the standard Poisson models assume independence of response conditional on covariates, which in the real world may not hold as a result of unobserved variables or shared latent processes. To address this, various extensions have been proposed, including those involving random effects or latent variables to capture correlation.

Earlier fertility models implemented in the studies above and similar ones not captured here typically addressed correlated reproductive outcomes indirectly through stratification, sequential modeling, or composite indicators, thereby failing to formally capture cross‐outcome dependence. Multivariate count models overcome these limitations by jointly modeling the CHD, TCB, NLC, and their associated independent variables within a unified framework that explicitly accounts for shared unobserved reproductive factors. While such models rely on distributional assumptions and cannot correct systematic reporting biases, they provide a more faithful representation of the reproductive process and offer substantial gains in efficiency, interpretability, and inferential validity.

Although comprehensive joint modeling of CHD, TCB, and NLC under a single framework with advanced statistical techniques like a trivariate Poisson regression (TPR) model via the Expectation‐Maximization (EM) algorithm is not common, this study will assist in filling this gap by trying a more exhaustive and robust analysis. The TPR model is apt to this task because it allows each response to be modeled as a covariate function and adjusts for the common variability in terms of latent factors. The EM algorithm facilitates parameter estimation in this complex model structure by handling the missing latent effects well. The EM algorithm is a general iterative method for finding maximum likelihood parameter estimates in statistical models under conditions where the data contain latent variables or missing data [29]. Its application to demographic research has been largely in missing data scenarios or mixture models [30].

The EM algorithm has previously been used for estimating parameters in latent class models appearing in fertility preference or models dealing with interval‐censored failure time data. It can model correlated count outcomes jointly, especially when unobserved variables explain the correlation. The EM algorithm has been implemented to obtain parameter estimates in several bivariate Poisson regression models using the bivariate Poisson distribution [31, 32]. The maximum likelihood‐based estimation technique to obtain parameter estimates in the bivariate Poisson regression model provides interesting but challenging outcomes [27, 33, 34, 35]. These drawbacks emphasize how crucial it is to carefully assess and use an appropriate numerical technique for a given situation [36]. Particularly if the starting estimate is far from the real value, convergence problems may occur in the estimation, preventing it from converging to a solution [37]. If convergence is achieved, it can lead to the convergence of the process to a local maximum as opposed to the global maximum [38]. This can cause oscillation or divergence, particularly in cases where the function exhibits non‐linear behavior, as in the case of the trivariate Poisson distribution and the regression model that goes along with it [39, 40, 41, 42].

Despite the possibility of requiring several iterations, traditional numerical techniques may converge slowly [33]. The choice of beginning values determines the convergence and precision of the method [37]. The process takes the function to be differentiable with a smooth curve; however, this may not be the case in practice. Traditional numerical techniques may therefore not be appropriate for all purposes. The estimating process may come to an endless loop when these traditional techniques produce a local maximum [37]. The EM method, in contrast to traditional techniques, is a potent parameter estimation instrument that comes with several advantages and some flexibility [43]. It handles missing data in various applications by using latent variables as a tool [44]. The EM algorithm is resilient to extremes and distortions in the data. There is a guarantee that the EM algorithm will eventually converge to the likelihood function's local maximum. Particularly for huge datasets, the EM algorithm can be more effective than conventional optimization methods [45].

The TPR model implemented is an extension of the trivariate Poisson distribution to include independent variables, taking into consideration their common covariance term. The study demonstrates a first attempt at utilizing the TPR model with model fitting by EM for the examination of correlated count data with potential unobserved heterogeneity. This provides an applied methodological contribution, which brings a robust methodology to analyzing similar multi‐outcome demographic data. The methodological framework developed in this study can serve as a model for future research that seeks to examine other interlinked count outcomes in demography and public health, aside from the specific variables examined in this study.

Through the identification of some of the key determinants of CHD, TCB, and NLC, the findings will provide evidence‐based policy recommendations to policymakers. This can guide the development of family planning‐related targeted interventions, maternal and child health programs, and education programs. The study findings on the relationship between cohabitation duration and fertility outcomes and child survival are applicable in informing public health interventions on reproductive health to promote healthier family planning practices and expanded access to basic health care services among women and children.

The data utilized in the analysis, the executed methods, the results of the analyses and their discussion, as well as the conclusions, are presented in the subsequent sections.

## 2 Materials and Methods

### 2.1 Data

The data used for the study were obtained from the 2022 Ghana Demographic and Health Survey (GDHS) [8]. The data was obtained after an official request was made and the request granted at https://dhsprogram.com/data/dataset/Ghana_Standard-DHS_2022.cfm?flag=0. The variables selected for use included the number of years since cohabitation (CHD), the total number of children (TCB), the number of living children (NLC), the number of births in the last 5 years (BF), the number of entries in the birth register (EBR), and the number of pregnancy losses (PL) of each woman involved in the study. The dependent variables included CHD, TCB, and NLC, while the remaining variables served as independent variables. The data included 15,014 women of reproductive age (15–49 years). Among the women involved in the study, forty percent (40%) were married, whereas fifteen percent (15%) were in relationships likened to marriage [8]. Eight percent (8%) were divorced or separated [8]. Thirty‐five percent (35%) have never married, while the remaining proportion were widows [8].

The CHD variable is calculated based on the difference between the survey date and the date the woman reported first starting to live continuously with her husband or partner. It is used in analyses to assess the timing of life events, such as the cumulative percentage married by years since cohabitation began. To compute the TCB, DHS collects a full birth history from each woman surveyed, listing all live births with their dates. The DHS survey obligatorily records this number, and interviewers are not permitted to record “unknown” numbers of children. The total number of children ever born is derived from this birth history. If a woman has more than 20 births, the detailed birth history variables (listing up to 20 births) may be truncated, but still capture the full count. There is no theoretical limit to its entries. The NLC is derived from the full birth history collected from each woman and distinguishes children who are currently surviving from those who have died. DHS collects a complete birth history for each woman, recording dates of all live births and whether each child is still living. The variable is computed by summing all children in the birth history with a survival status of living.

The BF of women of reproductive age refers to all live births a woman aged 15–49 years has had in the period from 0 to 59 months before the month of the interview. This time frame is a standard reference period used to collect data on recent fertility, maternal, and child health indicators. The data collection specifically covers a period of 5 years (60 months) immediately preceding the start of the survey fieldwork for each respondent with information collected from individual women of reproductive age. The DHS questionnaire includes a detailed birth history section where women are asked about every live birth they have had, including the date of birth, sex, survival status, and age at death if the child died. The data from this history is then used to extract information on births within the specific 5‐year window. This specific timeframe is used to calculate various statistics related to maternal and child health. This measure is widely used in DHS reporting and analysis of recent fertility, maternal and child health outcomes, and health service utilization (e.g., antenatal care or delivery care) by focusing on births within a relatively recent and comparable recall period [8].

PL is the total count of all non‐live‐birth pregnancy outcomes a woman has experienced before the survey interview. This count is a self‐reported measure and includes spontaneous abortions (miscarriages), induced abortions, stillbirths, ectopic pregnancies,Molar pregnancies, and intrauterine fetal demise. Spontaneous abortions (miscarriages) are the loss of a pregnancy before the fetus can survive outside the womb, typically defined as before twenty (20) or twenty‐eight (28) weeks of gestation, depending on the specific study or local context, whereas an induced abortion is the intentional termination of a pregnancy. Stillbirths are the loss of a fetus after the viability threshold (e.g., after 28 weeks of pregnancy or with a birth weight of 1000 g or more), while ectopic pregnancies occur outside the uterus.

The exact definitions of these outcomes in DHS follow standard questionnaire coding: a pregnancy not ending in a live birth is treated as a loss in the reproductive calendar or full pregnancy history. The number of pregnancy losses can be tabulated over specific recall periods (e.g., 3 years preceding the survey in some DHS tables on pregnancy outcomes) or calculated for ever‐pregnant women if lifetime history is examined. The base population for this measure is usually women of reproductive age who have ever experienced a pregnancy. In analytical reporting, DHS data on miscarriages, induced abortions, and stillbirths are summed for each woman to derive the PL. The PL is a key reproductive health indicator used to assess patterns of adverse pregnancy outcomes and their determinants in populations. It is distinct from indicators such as live births in the last 5 years or the total number of children because it specifically focuses on pregnancies that did not result in surviving offspring.

The EBR refers to the number of birth records actually listed in the DHS birth history section for a woman. The birth register in the DHS is a component of the Women's Questionnaire, where interviewers collect a complete birth history from every eligible woman of reproductive age. This refers to the total quantity of live births a woman reports. The birth history is a comprehensive, event‐by‐event record where details for each child (sex, date of birth, survival status, age at death if applicable) are collected sequentially. Each child constitutes an “entry” or “occurrence” in this section of the data file. The term “register”, as used in the DHS query, does not refer to a formal government‐run civil registration system but rather to the internal data collection instrument (the birth history section) used within the DHS itself. It is capped at 20 births in standard DHS datasets. This is a data‐structure variable, not a fertility measure per se and may not be used in fertility analyses but can serve as a supporting variable. It essentially measures births with detailed records and is used as an index of available birth history data.

Standard demographic controls were excluded because the model conditions on detailed reproductive history measures, which directly capture exposure, timing, and intensity of childbearing and thus subsume the effects of age, marital status, education, wealth, and region. See Supporting Information S1: Table S for the full list of variable definitions.

The data were thoroughly cleaned before the analysis was conducted. Missing values were handled using multiple imputation by chained equations (MICE) under the assumption of missing at random [30, 46, 47], allowing all eligible women to be retained in the analytic sample. Internally consistent checks were performed to ensure agreement between summary and fertility measures and detailed birth history records. Implausible values were corrected or excluded before model estimation. All outcome variables were treated as non‐negative integer counts. Our analysis did not concentrate on making nationally representative estimates, but instead on the estimation of the dependence structure and the regression parameters of the suggested trivariate Poisson model. This means that the sampling weights that were part of the complex sample design used by the DHS were not included in the estimation process. Thus, our focus was on model‐based estimation through the use of maximum likelihood estimation for quantification of association among the reproductive events conditional on the covariates.

Missing data were assessed for all analytic variables, and the extent and patterns of missingness were examined before the analysis. To minimize bias and loss of efficiency due to item nonresponse, MICE was utilized for missing values. All analyses were conducted on the imputed datasets with results combined according to Rubin's rule [48]. Table 1 shows the summary statistics of the data used.

**Table 1:** Descriptive statistics of the data.

Statistic	CHD	TCB	NLC	BF	EBR	PL
Sample size	15,014	15,014	15,014	15,014	15,014	15,014
Mean	16.313	4.630	4.257	0.929	5.026	0.396
Standard deviation	4.062	2.233	1.997	0.842	2.350	0.767
Minimum	0.000	1.000	0.000	0.000	1.000	0.000
First quartile	11.000	3.000	3.000	0.000	3.000	0.000
Median	17.000	4.000	4.000	1.000	5.000	0.000
Third quartile	23.000	6.000	6.000	1.000	7.000	1.000
Maximum	37.000	13.000	11.000	4.000	14.000	11.000

## 3 Methods

Let $\left(Y_{1},Y_{2},Y_{3}\right)$ be three count outcomes (CHD, NLC, TCB) such that $Y_{1}=X_{4}+X_{1}$, $Y_{2}=X_{4}+X_{2}$, and $Y_{3}=X_{4}+X_{3}$, where $X_{4}\sim \mathrm{Poisson}(\Lambda_{4})$, $X_{1}\sim \mathrm{Poisson}(\Lambda_{1}-\Lambda_{4})$, $X_{2}\sim \mathrm{Poisson}(\Lambda_{2}-\Lambda_{4})$, $X_{3}\sim \mathrm{Poisson}(\Lambda_{3}-\Lambda_{4})$, $\Lambda_{4}\geq 0;$ $\Lambda_{j}>0,\Lambda_{j}>\Lambda_{4},j=1,2,3$. As a result, $Y_{1}\sim \mathrm{Poisson}(\Lambda_{1})$, $Y_{2}\sim \mathrm{Poisson}(\Lambda_{2})$, and $Y_{1}\sim \mathrm{Poisson}(\Lambda_{3})$. $X_{4}$,$X_{1}$,$X_{2}$ and $X_{3}$ are independent mutually. This implies that $E\left(Y_{j}\right)=\text{var}\left(Y_{j}\right)=\Lambda_{j},j=1,2,3$ and ${\text{cov}\left({Y_{i},Y}_{j}\right)=\Lambda }_{4},\;j=1,2,3;i=1,2,3;i\neq j$, is the common shared constant covariance [39, 40, 41, 45, 49, 50]. In the event that $\Lambda_{4}=0$, $Y_{1},Y_{2},{\text{and}\;Y}_{3}$ are deemed independent [39, 40, 41]. The probability mass function of the trivariate Poisson distribution with observed counts $y_{1},y_{2},y_{3}$ corresponding to $Y_{1},Y_{2},Y_{3}[$ 39, 40, 41], respectively is

(1) $$P\left(Y_{1}=y_{1},Y_{2}=y_{2},Y_{3}=y_{3}\right)=\left[\text{exp}-\left(\sum_{j=1}^{3}\Lambda_{j}-2\Lambda_{4}\right)\right]\sum_{k=0}^{\omega }\frac{\Lambda_{4}^{k}}{k!}\prod_{j=1}^{3}\frac{{\left(\Lambda_{j}-\Lambda_{4}\right)}^{y_{j}-k}}{(y_{j}-k)!}\;$$

$$\omega =\min\left(y_{1},y_{2},y_{3}\right)$$

i=1,2,3,…,n;j=1,2,3

Covariates (PL, EBR, BF) are incorporated into the model by introducing them through the $\Lambda_{j}(j=1,2,3)$ via a log‐link function as

(2) $$\Lambda_{j}=\text{exp}\left(z^{T}\gamma_{j}\right)$$

*z* is the matrix of covariates and $\gamma_{j}$ vector of parameter estimates, j=1,2,3. Since the shared common variance is of essence in this study, it is assumed that $\Lambda_{4}>0.$ Substituting Equation (2) into Equation (1) yields the conditional TPR model given by

(3) $$P\left(Y_{1}=y_{1},Y_{2}=y_{2},Y_{3}=y_{3}|z_{1},z_{2},\ldots ,z_{p}\right)=\left[\text{exp}-\left(\sum_{j=1}^{3}\text{exp}\left(z^{T}\gamma_{j}\right)-2\Lambda_{4}\right)\right]\sum_{k=0}^{\omega }\frac{\Lambda_{4}^{k}}{k!}\prod_{j=1}^{3}\frac{{\left(\text{exp}\left(z^{T}\gamma_{j}\right)-\Lambda_{4}\right)}^{y_{j}-k}}{(y_{j}-k)!}$$

The likelihood function under the observed count data utilizing Equation (3) is of the form

(4) $$L=\prod_{i=1}^{n}\left[\left[\text{exp}-\left(\sum_{j=1}^{3}\text{exp}\left(z^{T}\gamma_{j}\right)-2\Lambda_{4}\right)\right]\sum_{k=0}^{D}\frac{\Lambda_{4}^{k}}{k!}\prod_{j=1}^{3}\frac{{\left(\text{exp}\left(z^{T}\gamma_{j}\right)-\Lambda_{4}\right)}^{y_{\mathrm{ji}}-k}}{(y_{\mathrm{ji}}-k)!}\right]$$

$$D=\min\left(y_{1i},y_{2i},y_{3i}\right)$$

The log‐likelihood of TPR model is obtained by taking the natural logarithm of both sides of Equation (4). This yields

(5) $$l=\ln\left[\prod_{i=1}^{n}\left[\text{exp}-\left(\sum_{j=1}^{3}\text{exp}\left(z^{T}\gamma_{j}\right)-2\Lambda_{4}\right)\right]\sum_{k=0}^{D}\frac{\Lambda_{4}^{k}}{k!}\prod_{j=1}^{3}\frac{{\left(\text{exp}\left(z^{T}\gamma_{j}\right)-\Lambda_{4}\right)}^{y_{\mathrm{ji}}-k}}{(y_{\mathrm{ji}}-k)!}\right]$$

The Newton–Raphson procedure is particularly effective when the initial point is located in the vicinity of the maximum [51]. However, for a singular Hessian, no solution can be obtained [51]. As a consequence of this, the EM algorithm is utilized to overcome these hurdles and other complexities that arise in parameter estimation in the novel TPR model.

In the EM algorithm, the pseudo‐values are computed using the present estimates obtained in the nth iteration. These pseudo‐values are used to obtain the maximum lower bound on the log‐likelihood to get the new setting estimates, as well as iterations within these two steps until convergence is reached [45, 49, 50]. The algorithm is simplified in two steps: the expectation step (E‐step) and the maximization step (M‐step). In the E‐step, one estimates the expected value of the full data log‐likelihood function based on the observed data and current parameter values from the last iteration [45, 49, 50]. In the trivariate Poisson case, this involves estimating the expected values of the latent variables given the observed and current parameters. The calculated expected log‐likelihood function from the E‐step is maximized in the M‐step to obtain the regression coefficients and model parameters. Maximization typically results from solving equations derived by equating the derivatives of the expected log‐likelihood to zero [45, 49, 50]. The procedure for applying the EM algorithm to the TPR model is outlined below.

For each observation i=1,2,…,N, we have trivariate observed data $\left(Y_{1i},Y_{2i},Y_{2i}\right)$. Unobserved is the latent variable $X_{4i}$. The complete data for observation i would then be both the observed counts and the latent variable: $\left(Y_{1i},Y_{2i},Y_{2i},X_{4i}\right)$.

With the generative model given, $Y_{1i}=X_{1i}+X_{4i},Y_{2i}=X_{2i}+X_{4i},\text{and}\;Y_{3i}=X_{3i}+X_{4i}$, where $X_{1i},X_{2i},X_{2i},$ and $X_{4i}$ are mutually independent Poisson variables with respective rates $X_{4i}\sim \mathrm{Poisson}\left(\Lambda_{4}\right),$ $X_{3i}\sim \mathrm{Poisson}(\Lambda_{3i}-\Lambda_{4})$, $X_{2i}\sim \mathrm{Poisson}(\Lambda_{2i}-\Lambda_{4})$, and $X_{1i}\sim \mathrm{Poisson}(\Lambda_{1i}-\Lambda_{4})$ mutually independent such that $Y_{1i}\sim \mathrm{Poisson}(\Lambda_{1i}+\Lambda_{3i})$ and $Y_{2i}\sim \mathrm{Poisson}(\Lambda_{2i}+\Lambda_{3i})$ and $\mathrm{Cov}\;\left(Y_{\mathrm{ij}},Y_{\mathrm{ji}}\right)=\Lambda_{4},i=1,2,3;j=1,2,3\mathrm{;i}\neq j$. $\Lambda_{1i}>0,\Lambda_{2i}>0,$ $\Lambda_{3i}>0,\Lambda_{1i}>\Lambda_{4}$, $\Lambda_{2i}>\Lambda_{4}$ and $\Lambda_{3i}>\Lambda_{4}$. The $X_{1i},X_{2i},X_{3i}$ values could be learned from the full data: $X_{1i}=Y_{1i}-X_{4i},X_{2i}=Y_{2i}-X_{4i}$ and $X_{3i}=Y_{3i}-X_{4i}$.

The complete‐data log‐likelihood of a single observation i, under the independence of $X_{1i},X_{2i},X_{3i}$, and $X_{4i}$, can be expressed as in terms of the sum of the log‐likelihoods of the four independent Poisson distributions:

$L_{c}\left(\emptyset_{i}|Y_{1i},Y_{2i},Y_{2i},X_{4i}\right)=\ln P\left(Y_{1i}-X_{4i}|\Lambda_{1i},\Lambda_{4i}\right)+\ln P\left(Y_{2i}-X_{4i}|\Lambda_{2i},\Lambda_{4i}\right)+\ln P\left(Y_{3i}-X_{4i}|\Lambda_{3i},\Lambda_{4i}\right)+\ln P\left(X_{4i}|\Lambda_{4}\right)$

where $\emptyset_{i}=\left(\Lambda_{1i},\Lambda_{2i},\Lambda_{3i},\Lambda_{4}\right)$ are the parameters for the i−th observation, with $\Lambda_{1i},\Lambda_{2i},\Lambda_{3i}$ connected with the covariates $z_{i}$ via the log‐linear model and $\Lambda_{4i}$ the constant covariance relating to the latent factor. The complete‐data log‐likelihood for the entire N observations is the sum of these individual contributions for all N observations [49, 50, 52, 53, 54].

### 3.1 E‐Step (Expectation)

The objective of the E‐step at iteration (k+1) is to enumerate the expected estimate of the complete‐data log‐likelihood, conditioned on the data observed and $\emptyset^{\left(k\right)},$ the recent estimates of parameter. This expected value is typically denoted as $Q\left(\emptyset |\emptyset^{\left(k\right)}\right)[$ 49, 50, 52, 53, 54].

This step is essentially the computation of the conditional expectation of the latent variables $X_{4i}$ for every observation, conditioned on the observed counts $\left(Y_{1i},Y_{2i},Y_{2i}\right)$ and the current parameter estimates $\emptyset_{i}^{\left(k\right)}$. In particular, the desired quantity is E. The conditional distribution of $X_{4i}$ given $\left(Y_{1i},Y_{2i},Y_{2i}\right)$ is proportional to the product of the Poisson probability mass functions (pmfs):

$$P\left(Y_{\mathrm{ji}},X_{4i}|\Lambda_{1i},\Lambda_{2i},\Lambda_{3i},\Lambda_{4}\right)=P\left(Y_{1i}-X_{4i}|\Lambda_{1i}\right)P\left(Y_{2i}-X_{4i}|\Lambda_{2i}\right)P\left(Y_{3i}-X_{4i}|\Lambda_{3i}\right)P\left(X_{4i}|\Lambda_{4}\right)$$

This conditional distribution is usually non‐standard and requires summation over potential values of latent factor $\left(0,\min(y_{1i},y_{2i},y_{3})\right)$ to compute the normalizing constant and expectation. The derived expectation E will subsequently be utilized as a pseudo‐value or sufficient statistic in the following M‐step [49, 50, 52, 53, 54].

### 3.2 M‐Step (Maximization)

The role of the M‐step is to amplify the mean complete‐data log‐likelihoods, $Q\left(\emptyset |\emptyset^{\left(k\right)}\right)$, concerning the parameters $\emptyset =\left(\gamma_{1},\gamma_{2},\gamma_{3},\gamma_{4}\right)$. One of the advantages of the EM algorithm in such cases is that due to the additive nature of the complete‐data log‐likelihood (which will be an aggregate of independent Poisson log‐likelihoods), this maximization will be decoupled into separate generalized linear model (GLM) problems [49, 50, 52, 53, 54].

For example, to re‐estimate the regression coefficients $\gamma_{1}$, terms in $Q\left(\emptyset |\emptyset^{\left(k\right)}\right)$ containing $\Lambda_{1i}$ are maximized. Plugging in the expected value of $X_{4i}$ (from the E‐step), this becomes a standard Poisson regression issue with response (or the same, depending on the exact derivation of sufficient statistics), and with log‐link function connecting $\Lambda_{1i}$ to the covariates $z_{i}$. The same Poisson regressions would be performed to obtain updated estimates for $\gamma_{2}$ and $\gamma_{3}$.

Likewise, for the $\gamma_{4}$ (those that regulate $\Lambda_{4i}$), the maximization would be over terms that include $X_{4i}$. This typically collapses to a standard Poisson distribution where the expected value of $X_{4i}$, and its rate $\Lambda_{4i}$ is a constant. The M‐step thus supplies the updated parameter estimates $\emptyset^{\left(k\right)}$.

The EM algorithm begins with a starting guess for the parameters, $\emptyset^{\left(0\right)}$. Then it repeatedly alternates between the E‐step and the M‐step, computing a sequence of estimates for the parameters:

$\emptyset^{\left(0\right)},\emptyset^{\left(1\right)},\emptyset^{\left(3\right)}$

One of the key properties of the EM algorithm is that it will guarantee the observed data log‐likelihood to be non‐decreasing at each successive iteration, and it makes progress towards a local maximum [49, 50, 52, 53, 54].

Convergence of the algorithm is typically verified by monitoring the change in the log‐likelihood value or the estimates of the parameters between successive iterations [49, 50, 52, 53, 54]. Convergence of the algorithm is stated to materialize, albeit the absolute divergence in the log‐likelihood or Euclidean distance of vectors of parameters is less than a predetermined small number, ϵ

$$│\max\left(\emptyset^{\left(t+1\right)}\;\right)-\max\left(\emptyset^{\left(t\right)}\;\right)│\leq \epsilon ,\;\epsilon >0$$

The decoupling ability of the EM algorithm and its integration with generalized linear models (GLMs) are the major advantages. The algorithm, by effective imputation of the absent latent data (in expectation), transforms the difficult trivariate problem into a series of simple, independent univariate Poisson regression (UPR) problems at the M‐step. This means that high‐quality existing GLM packages available can be applied directly to the maximization step, greatly reducing the practitioners' programming effort and computation sophistication. This is a powerful practical benefit, demonstrating the way that the EM algorithm bridges the gap between sophisticated theoretical models and available computing capabilities [49, 50, 52, 53, 54].

The EM algorithm has its origins actually in the missing data principle. For the TPR model, the latent common variable $X_{4i}$ is not missing as an error in data collection, but is unobserved intentionally as part of the model's generative process. This redefinition of an unobserved subset as missing data is a central conceptual advance that makes the use of the EM algorithm possible. It enables the application of a robust, general model parameter estimation framework when some variables cannot be measured directly but are surely included in a manner to fully specify the joint distribution and underlying dependence structure. This perspective is deeper in appreciating the estimation of latent variable models and underscores the wide applicability of the EM algorithm.

The steps for the EM algorithm for the proposed conditional TPR model implemented in R is as follows:

1. Define the TPR model with covariates utilizing Equation (3). The latent variable $X_{4}$ remains a Poisson variable, typically with a constant parameter $\Lambda_{4}$. The parameters to be estimated are now $\gamma_{1},\gamma_{2},\gamma_{3},\text{and}\;\gamma_{4}$. Let $\emptyset =\left(\gamma_{1},\gamma_{2},\gamma_{3},\gamma_{4}\right)$.
2. Initialize the parameters at $\emptyset^{\left(t\right)}$ at some starting values for t=0.
3. This step remains largely the same as the previous algorithm, but with the log‐linear parameterization. For each observation i, the conditional expectation of $X_{4}$, latent variable, E, is calculated given the observed data $y_{i}$, the covariates $z_{i}$, and the current parameter estimates $\emptyset^{\left(t\right)}$.

$$E\left[X_{4i}|y_{i},z_{i},\emptyset^{\left(t\right)}\right]=\sum_{k=0}^{D}\mathrm{kP}\left({k=X}_{4i}|y_{i},z_{i},\emptyset^{\left(t\right)}\right)$$

where,

$$P\left(y_{\mathrm{ji}},k{z_{i},\emptyset }^{\left(t\right)}\right)=P\left(y_{1i}-k\Lambda_{1i}^{\left(t\right)}\right)P\left(y_{2i}-k\Lambda_{2i}^{\left(t\right)}\right)P\left(Y_{3i}-k\Lambda_{3i}^{\left(t\right)}\right)P\left(k\Lambda_{4}^{\left(t\right)}\right)$$

$$P\left(y_{\mathrm{ji}}|{z_{i},\emptyset }^{\left(t\right)}\right)=\sum_{k=0}^{D}P\left(y_{\mathrm{ji}},k{z_{i},\emptyset }^{\left(t\right)}\right)$$

$$\Lambda_{\mathrm{ji}}^{\left(t\right)}=\text{exp}\left(z^{T}\gamma_{j}\right),j=1,2,3$$

$$D=\min(y_{1i},y_{2i},y_{3i})$$

4. The update rules for $\gamma_{j}(j=1,2,3)$, henceforth have no simple closed form resulting from the log‐linear link function. The update for $\gamma_{4}$ remains the same. The M‐step involves maximizing the expectation of the complete‐data likelihood $Q(\emptyset |\emptyset^{\left(t\right)})$, concerning the new parameters.

Update for $\gamma_{4}$:

The update rule for is straightforward:

$$\gamma_{4}=\frac{1}{n}\sum_{i=1}^{n}E\left[X_{4i}|y_{i},z_{i},\emptyset^{\left(t\right)}\right]$$

Updates for $\gamma_{j}\;(j=1,2,3)$:

$$Q\left(\gamma_{1},\emptyset^{\left(t\right)}\right)=\sum_{i=1}^{n}\left\{\left(y_{1i}-E\left[X_{4i}|y_{i},z_{i}{,\emptyset }^{\left(t\right)}\right]\right)\text{exp}\left(z_{i}^{T}\gamma_{1}\right)-\text{exp}\left(z_{i}^{T}\gamma_{1}\right)\right\}+W$$

Wis a constant

Then

$$\frac{\partial Q\left(\gamma_{1},\emptyset^{\left(t\right)}\right)}{\partial \gamma_{1}}=\sum_{i=1}^{n}z_{i}\left\{\left(y_{1i}-E\left[X_{4i}|y_{i},z_{i},\emptyset^{\left(t\right)}\right]\right)\text{exp}\left(z_{i}^{T}\gamma_{i}\right)-\text{exp}\left(z_{i}^{T}\gamma_{1}\right)\right\}$$

Maximizing this objective function requires an iterative method as there is no closed‐form solution. The Newton–Raphson algorithm is utilized for this task. The updates for $\gamma_{2}$ and $\gamma_{3}$ are performed similarly.

5. The algorithm iterates between the E‐step and M‐step until the change in the parameters or the data observed log‐likelihood falls below a specified tolerance level, ϵ, is a small positive integer. The convergence condition is

$$│\max\left(\emptyset^{\left(t+1\right)}\;\right)-\max\left(\emptyset^{\left(t\right)}\;\right)│\leq \epsilon ,\;\epsilon >0$$

Initial values for outcome‐specific Poisson intensity parameters were obtained from separate univariate Poisson regressions fitted to each outcome. In contrast, the shared latent covariance parameter was initialized at a small positive value to reflect weak but non‐zero dependence across outcomes. Convergence was assessed using relative changes in the log‐likelihood and parameter estimates. Robustness to initialization was evaluated by repeating the EM algorithm with alternative starting values for the shared covariance parameter and regression coefficients; in all cases, the algorithm converged to the same solution, indicating stability of the likelihood surface.

To verify how good the EM performed in the regression, the standard errors of the parameter estimates were obtained [55]. The expression for this is

$$\mathrm{Standard}\;\mathrm{error}=\sqrt{{\left[I^{-1}\left(\hat{\gamma }\right)\right]}_{\mathrm{ii}}}$$

$I^{-1}\left(\hat{\gamma }\right)$ is the inverse of the observed Fisher information, which is the negation of the Hessian evaluated at $\hat{\gamma }.$ ${\left[.\right]}_{\mathrm{ii}}$ is the ith diagonal element corresponding to the ith parameter estimate.

Standard error values closer to 0 indicate a good fit for the parameter, whereas large values show a poor fit. The model goodness‐of‐fit of the model is assessed using the Akaike information criterion (AIC) and the Bayesian information criterion (BIC). The expressions for these are

AIC=−2l+2m

BIC=−2l+2mlogn

l is the log‐likelihood, m the number of parameters in the model, and n the size of the sample.

The efficiency of the overall model fit is checked utilizing the likelihood ratio test [56, 57, 58]. To verify the significance of the model, let assume the following hypothesis

$H_{0}:\gamma_{i}=0\left(\mathrm{no covaraite is significant in the model}\right)\;$

$H_{1}:\gamma_{i}\neq 0(i.e.\mathrm{at least one of the three covariates is significant})$

The likelihood ratio test statistic is given by

$$ℵ=\frac{L\left(\gamma_{i}=0\right)}{L\left(\gamma_{i}\neq 0\right)}$$

Where $L\left(\gamma_{i}=0\right)$ is the likelihood of the trivariate Poisson regression model evaluated without considering any covariate in the model. $L\left(\gamma_{i}\neq 0\right)$ is the likelihood of the trivariate Regression model evaluated by including all three covariates in the model (i.e., BF, EBR, PL).

Due to the asymptotic normality of the estimate based on the maximum likelihood [56, 57, 58], we have

(6) $$-2\ln ℵ=-2\ln\left[\frac{L\left(\gamma_{i}=0\right)}{L\left(\gamma_{i}\neq 0\right)}\right]\sim \chi_{\mathrm{df},0.95}^{2}\;\;\;\;\;\;\;\;\;\;$$

Where df is the degrees of freedom associated with the test, which is the same as the total number of covariates included in the trivariate Poisson regression model. The level of significance is 0.05.

Simplifying Equation (6) yields

$$-2\ln ℵ=2\left(l_{1}-l_{0}\right)\;$$

Where $l_{1}=\ln\left[L\left(\gamma_{i}\neq 0\right)\right]$ and $l_{0}=L\left(\gamma_{i}=0\right)$.

In the hypothesis test above, $H_{0}$ is rejected if $2\left(l_{1}-l_{0}\right)\geq \chi_{\mathrm{df},0.95}^{2}$. As a result, the p‐value of the test can be computed as $P\left[2\left(l_{1}-l_{0}\right)\geq \chi_{\mathrm{df},0.95}^{2}\right]$. Hence, $H_{0}$ is rejected if $P\left[2\left(l_{1}-l_{0}\right)\geq \chi_{\mathrm{df},0.95}^{2}\right]\leq 0.05$. When $H_{0}$ is rejected, the model is said to be significant, and at least one covariate is substantial in it. However, if the test fails to reject $H_{0}$, then none of the covariates is substantial in the model. In that instance, no covariate is needed in the model.

The Wald test is utilized to find those significant covariates among the three covariates included in the model for specific interpretability and policy direction [58, 59, 60, 61].

The procedure is implemented in R version 4.2.2, and the results are presented and discussed in the next section.

## 4 Results and Discussion

**Figure 1:**
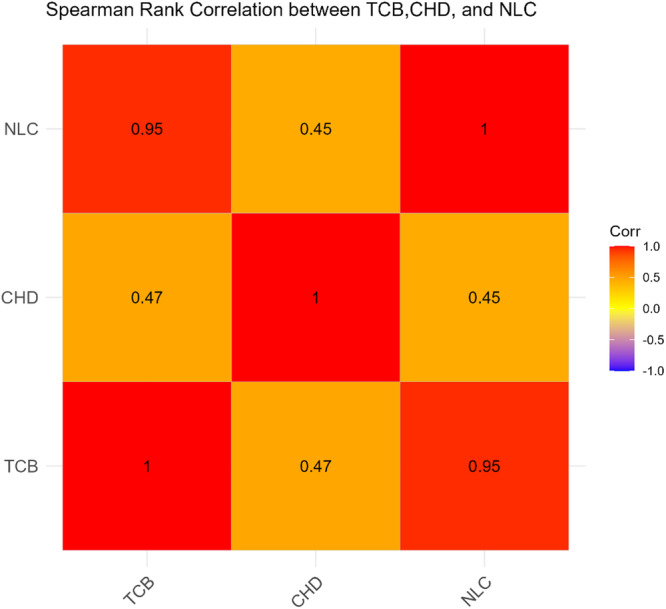
Spearman's rank correlation plot for the total number of children ever born (TCB), the number of years since cohabitation (TCB), and the number of living children (NLC).

Figure 1 shows the Spearman's rank correlation plot, illustrating the dependency between the dependent variables (i.e., TCB, NLC, and CHD). The results show a positive correlation between any two of the dependent variables selected. This confirms a satisfactory condition for implementing the trivariate Poisson regression model [39, 40, 41, 42, 45, 49, 62]. The results indicate a strong reciprocity between the number of living children and the total number of children ever born to a woman of reproductive age. This affirms the higher propensity of the women to have more living children with more births [63]. There is a relatively moderate interdependence between the total number of children born to a woman and the number of years of cohabitation. A similar, relatively moderate interdependency is observed between the total number of living children a woman has and the number of years of cohabitation. This shows that the number of living children and the total number of children born to a woman are moderately associated with the number of years of cohabitation with her partner [3, 64].

To assess the overall significance of the trivariate Poisson regression model, the likelihood ratio test was conducted [56, 57, 60], and the results are shown in Table 2. At a level of significance of 0.05, the likelihood ratio test indicates the model is significant. This is a revelation that at least one independent variable is significant in the model [56, 57, 60]. To ascertain which of the independent variables are significant in the model, the Wald test is implemented [58, 59, 60, 61]. The model's goodness‐of‐fit is shown in Table 3. Table 4 shows the results of the overall model with individual parameter estimates and the *p* values. The significance level is set at 0.05.

**Table 2:** Likelihood ratio test results.

Statistic	Estimate
Log‐likelihood (null):	−125,424.00
Log‐likelihood (full)	−112,598.50
LRT statistic	25,651.03
Degrees of freedom	9
*p* value	< 0.001

**Table 3:** Goodness‐of‐fit for the TCB, CHD, and NLC joint model.

Goodness‐of‐fit	Estimate
Log‐likelihood (full)	−112,598.50
AIC	225,223.00
BIC	225,322.10

**Table 4:** Parameters and standard errors for trivariate Poisson regression model of TCB, CHD, and NLC via the EM algorithm.

Dependent variable	Parameter	Estimate	Standard error	*p* value
TCB	Intercept	−1.5106	0.0442	< 0.001
	BF	−0.0676	0.0109	< 0.001
	EBR	0.3636	0.0042	< 0.001
	PL	−0.3584	0.0146	< 0.001
CHD	Intercept	2.2687	0.0063	< 0.001
	BF	−0.2468	0.0031	< 0.001
	EBR	0.1057	0.0010	< 0.001
	PL	−0.0812	0.0032	< 0.001
NLC	Intercept	−1.7973	0.0565	< 0.001
	BF	−0.0239	0.0130	0.066
	EBR	0.3613	0.0052	< 0.001
	PL	−0.3549	0.0182	< 0.001
	Shared covariance (standard error)	3.0101 (0.0117)		

In the model with TCB as the dependent variable, the intercept, BF, EBR, and PL were all significant at 0.05 significance level. If BF, EBR, and PL are held constant, the expected TCB will be reduced by 77.92%. This illustrates that for a fixed number of entries in the birth register, pregnancies in the last 5 years, and pregnancy losses, the total number of children born to the woman will be reduced by 77.92%. Thus, a woman of reproductive age who does not get pregnant in her last 5 years or does not register any births will have her expected fertility rate reduced by 77.92%. This result confirms the study by [24]. For a fixed EBR and PL, an extra BF will reduce the expected TCB by 6.54%. This is an indication that for any additional birth by a woman of reproductive age, the possible total number of children she intends to have reduces by 6.54%. A result that affirmed the studies by [65]. When BF and PL are kept constant, an additional EBR increases the expected TCB by 43.54%. Just as depicted by [66], any additional registration of birth by a woman increases her total number of children ever born by 43.54%. Similarly, for a constant BF and EBR, an additional PL decreases the expected TCB by 30.12%. This is an indication that for any pregnancy loss by a woman of reproductive age, the total expected number of children she intends to have reduces by 30.12%. This aligns with the results of [5].

Again, for the model with CHD as the dependent variable, the intercept, BF, EBR, and PL were all significant at the 0.05 significance level. If BF, EBR, and PL are held constant, the expected CHD will increase by 866.68%. This clearly shows that the expected number of years of cohabitation by women of reproductive age is extremely high when no births are registered or no pregnancies are recorded. A confirmation of the study conducted by [67]. An additional BF reduces the expected CHD by 21.87% if EBR and PL are held constant. This indicates that any extra birth by a woman of reproductive age in her last 5 years reduces her number of years of cohabitation by 21.87%, confirming studies by [25, 66]. A unit addition in EBR increases the expected CHD by 11.15%. Thus, any time a woman of reproductive age registers her birth, her expected years of cohabitation increase by 11.15% [68]. For any additional PL, the expected CHD decreases by 7.80% when BF and EBR are constant. This confirms that the expected number of years of cohabitation by women of reproductive age is reduced by 7.80% for any pregnancy loss. A result similar to that of [5, 69].

In the NLC model, the intercept, EBR, and PL were significant, while BF was not significant at the 0.05 significance level. This empirically shows that the number of living children of a reproductive woman does not statistically depend on her pregnancies in her last 5 years. When all other conditions (EBR and PL) remained constant, the expected NLC decreased by 83.43%. A revelation that if a woman of reproductive age lives a good and well‐balanced life, her fertility rate is expected to decrease significantly [66, 70]. For any additional birth registered by a woman of reproductive age, the expected NLC increases by 43.52% if all other factors remain constant. However, any additional PL decreases NLC by 29.88% when all other conditions remain stable. A similar result is conveyed by [66, 70].

In effect, a woman's years of cohabitation and fertility rate are influenced by the number of pregnancies in her last 5 years, the number of pregnancy losses, and the number of births registered, while her number of living children depends on the number of pregnancy losses and the number of births registered.

Fertility, pregnancy outcomes, and birth registration are central indicators used to monitor maternal health, reproductive behavior, and civil registration systems. The modeling of these correlated reproductive outcomes accounts for shared unobserved reproductive heterogeneity that influences multiple indicators simultaneously. The approach improves statistical precision, avoids biased influence, and aligns methods with real‐world reproductive processes. The integrated model here provides clearer insights into fertility dynamics and better reflects women's reproductive life course as captured in DHS data. It provides evidence on women's reproductive trajectories, autonomy, and access to family planning. It supports the monitoring and strengthening of civil registration and vital statistics (CRVS) systems by linking fertility outcomes with birth registration. This approach supports coordinated strategies across maternal health, family planning, and birth registration, as well as strengthens results‐based frameworks and Sustainable Development Goal reporting using internally consistent indicators.

The integrated modeling of correlated reproductive outcomes using the DHS data provides stronger, more actionable evidence to guide policies advancing maternal health, gender equality, and universal legal identity.

## 5 Conclusion

This probe provides a novel application of the trivariate Poisson regression model for the concurrent analysis of three correlated reproductive indicators: years of cohabitation, total number of children born, and number of surviving children, among women of reproductive age. This exploration contributes to demographic modeling by employing a trivariate Poisson regression with the EM algorithm to simultaneously analyze years of cohabitation, fertility, and child survival among women of reproductive age. Data from the 2022 Ghana Demographic and Health Survey were utilized. The findings validate the strong interrelation among cohabitation, fertility, and child survival, emphasizing the inadequacy of analyzing these outcomes separately. The proposed model accurately accounts for latent dependencies due to unobserved heterogeneity, and the EM algorithm ensures convergence and stability where past methods may fail. The implications highlight how reproductive patterns are shaped by factors such as recent fertility histories, birth registration practices, and pregnancy loss, lending an empirical foundation to evidence‐based interventions in maternal and child health. The joint modeling approach provides a framework for future public and population health research that seeks to capture the full intricacies of multiple count‐based outcomes. Government must integrate monitoring of fertility, child survival, and marriage patterns into health policy and enhance access to and utilization of birth registration services. The government and its stakeholderds must enhance family planning and fertility counseling services, address pregnancy loss through targeted maternal health interventions, and promote reproductive health education to delay early cohabitation. Fertility awareness initiatives and cohabitation duration should be incorporated into reproductive health programs.

The study has some limitations. Firstly, though the data used in the analyses was from the nationally representative 2022 Ghana Demographic and Health Survey, the analyses did not account for DHS sampling weights and survey complexities. Since the aim of this study was methodological development and modeling inference instead of population‐level estimations, the trivariate Poisson regression model proposed was estimated by unweighted maximum likelihood estimation. As a result, while the estimated relationship and dependence structures are still relevant in understanding the association between the reproductive outcomes, the regression coefficients might not capture the estimates at the population level because of the survey complexities. Future studies should consider proposing a trivariate Poisson approach that can be generalized to survey‐weighted, stratified, and clustered data to estimate nationally representative parameters while maintaining the dependence structures. Secondly, implementing the EM algorithm in R takes a long time to converge; as a result, an alternative optimization algorithm that yields faster results could be considered in future studies. Also, alternative multivariate count frameworks (e.g., negative binomial or random‐effects specifications) may yield different dependence structures and remain areas for future studies.

## Author Contributions

**Kassim Tawiah:** conceptualization, writing – original draft, methodology, validation, visualization, writing – review and editing, data curation, formal analysis. **Nana Kena Frempong:** conceptualization, writing – original draft, methodology, validation, visualization, writing – review and editing, formal analysis, supervision, data curation. **Atinuke Olusola Adebanji:** conceptualization, writing – original draft, methodology, validation, visualization, writing – review and editing, formal analysis, supervision, data curation. **Richard Kwame Ansah:** conceptualization, validation, visualization, writing – review and editing, data curation.

## Funding

The authors have nothing to report.

## Ethics Statement

The 2022 Ghana Demographic and Health Survey (GDHS) obtained ethical clearance from the Ethics Review Committee of the Ghana Health Service. Additionally, the ICF Institutional Review Board also approved the GDHS ethically.

## Conflicts of Interest

The authors declare no conflicts of interest.

## Transparency Statement

The corresponding author, Kassim Tawiah, affirms that this manuscript is an honest, accurate, and transparent account of the study being reported; that no important aspects of the study have been omitted; and that any discrepancies from the study as planned (and, if relevant, registered) have been explained.

## Supporting information

Supporting File

## Data Availability

The data used in the study is available at https://dhsprogram.com/data/dataset/Ghana_Standard-DHS_2022.cfm?flag=0. The R codes used for the model implementation will be made available upon request from the corresponding author.
