# Supplementary material for: Modeling of Years of Cohabitation, Children Ever Born, and Number of Living Children of Women of Reproductive Age Using Trivariate Poisson Regression
Source: Health Sci Rep. 2026 Aug 16;9(8):e72982. doi: 10.1002/hsr2.72982 (PMC13478850; doi:10.1002/hsr2.72982)
Supplement: Supplementary file 1 [file HSR2-9-e72982-s001.docx]

| Variable | Definition |
| --- | --- |
| Years of cohabitation (CHD) | CHD refers to the **duration in years since a woman of reproductive age (typically 15-49 years) began living with a partner in a marriage-like relationship**, regardless of whether the union is formally married or consensually cohabiting |
| Total number of children (TCB) | TCB of women of reproductive age is to the **total number of live births a woman has ever had in her lifetime**, regardless of whether the children are currently alive or deceased |
| Living children (NLC) | NLC is the **total count of a woman's biological children who are alive at the time of the survey** |
| Births in the last five years (BF) | The BF of women of reproductive age refers to all live births a woman aged 15-49 years has had in the period from 0 to 59 months before the month of the interview |
| Entries in the birth register (EBR) | EBR refers to the **number of birth records actually listed in the DHS birth history section** for a woman |
| Pregnancy losses (PL) | PL is the total count of all non-live-birth pregnancy outcomes a woman has experienced before the survey interview |

Table S: Definition of variables used in the study
